# Aldose Reductase B1 in Pig Sperm Is Related to Their Function and Fertilizing Ability

**DOI:** 10.3389/fendo.2022.773249

**Published:** 2022-01-31

**Authors:** Yentel Mateo-Otero, Jordi Ribas-Maynou, Ariadna Delgado-Bermúdez, Marc Llavanera, Sandra Recuero, Isabel Barranco, Marc Yeste

**Affiliations:** ^1^ Unit of Cell Biology, Department of Biology, Faculty of Sciences, University of Girona, Girona, Spain; ^2^ Biotechnology of Animal and Human Reproduction (TechnoSperm), Institute of Food and Agricultural Technology, University of Girona, Girona, Spain; ^3^ Department of Veterinary Medical Sciences, University of Bologna, Ozzano dell’Emilia, Bologna, Italy

**Keywords:** aldose reductase B1, AKR1B1, epididymal maturation, ejaculated sperm, sperm physiology, *in vitro* fertilization (IVF)

## Abstract

Aldose reductase B1 (AKR1B1) has been reported to participate in the modulation of male and female reproductive physiology in several mammalian species. In spite of this, whether or not AKR1B1 could be related to sperm quality, functionality and fertilizing ability is yet to be elucidated. The present study, therefore, aimed to investigate: i) the presence of AKR1B1 in epididymal and ejaculated sperm; ii) the relationship between the AKR1B1 present in sperm and the physiology of the male gamete; iii) the liaison between the relative content of AKR1B1 in sperm and their ability to withstand preservation for 72 h; and iv) the potential link between sperm AKR1B1 and *in vitro* fertility outcomes. Immunoblotting revealed that AKR1B1 is present in both epididymal and ejaculated sperm with a similar relative content. Moreover, the relative levels of AKR1B1 in sperm (36 kDa band) were found to be negatively related to several kinematic parameters and intracellular calcium levels, and positively to the percentage of sperm with distal cytoplasmic droplets after storage. Finally, AKR1B1 amounts in sperm (36 kDa band) were negatively associated to fertilization rate at two days post-fertilization and embryo development at six days post-fertilization. The results of the present work suggest that AKR1B1 in sperm is probably acquired during maturation rather than at ejaculation and could play a role in that process. Moreover, AKR1B1 seems to be related to the sperm resilience to preservation and to their fertilizing capacity, as lower levels of the 36 kDa band (putative inactive form of this protein) result in better reproductive outcomes.

## Introduction

Predicting fertility remains a major challenge for reproductive biology in mammals (1), that is why a significant number of studies have focused on uncovering male fertility biomarkers in the last years (2–4). In this context, proteomic characterization of both seminal plasma [SP; (5, 6)] and sperm (7) has gained much relevance. Pérez-Patiño et al. (2018) performed an in-depth proteomic analysis of pig SP revealing that specific proteins were related to sperm fertilizing ability (8). Among these proteins, these authors identified Aldose Reductase B1 (AKR1B1 or ALR2), which is a NADPH-dependent enzyme that belongs to the aldo-keto reductase protein superfamily (9, 10) and is positively related to *in vivo* fertility outcomes (8).

Aldose Reductase B1 is the most studied aldose reductase and participates in both the polyol pathway and the detoxification of carbonyl compounds in many cells and tissues (11–13), including the male genital tract (14, 15). This protein has been reported to be involved in both male and female reproductive physiology in several mammalian species, including humans (16), cattle (17, 18), rats (15), sheep (19) and pigs (8, 20, 21). Focusing on the male, while AKR1B1 in SP has been reported to exert a positive effect on *in vivo* fertility outcomes in porcine (8), it does not seem to influence sperm physiology in terms of sperm survival and motility, intracellular H_2_O_2_ levels, acrosome integrity and plasma membrane stability (21). Not only is AKR1B1 present in SP but also in sperm, where it appears to be involved in epididymal maturation through the polyol pathway for fructose production (17, 22, 23). In addition, AKR1B1 is activated during sperm capacitation and modulates sperm motility, probably through balancing reactive oxygen species (ROS) production (24). Yet, whether does sperm AKR1B1 modulate other sperm functional parameters or influence the sperm ability to fertilize the oocyte has not been investigated.

Although aldose reductases have been identified in bovine (17, 25), equine (26) and porcine sperm (24), the origin of this protein in mature sperm cells is still unknown. In this regard, while translation during spermatogenesis could be a possibility, no previous study has confirmed if the relative content of AKR1B1 is higher in ejaculated than in epididymal sperm. In bovine sperm, the relative amount of this protein has been found to increase along the epididymal transit (17, 25), probably due to the integration of epididymosomes at the cauda (25). In addition, bovine prostasomes have been reported to contain AKR1B1 (22). For this reason, determining whether the relative content of AKR1B1 is higher in ejaculated than in epididymal sperm would provide further evidence on the aforementioned contribution of the extracellular vesicles present in SP.

Considering the relevance of AKR1B1 as a vital protein in mammalian reproductive physiology, the main aim of the present study was to determine the potential involvement of sperm AKR1B1 in both sperm physiology and fertility outcomes using the pig as a model. The following specific objectives were set: (1) to identify the presence of AKR1B1 in epididymal and ejaculated sperm in order to elucidate whether this protein is acquired from SP upon ejaculation; (2) to assess the relationship between sperm AKR1B1 and the physiology of male gametes; (3) to determine whether sperm AKR1B1 is related to the sperm resilience to preservation in liquid storage; and (4) to evaluate the relationship between the relative content of AKR1B1 in sperm and their *in vitro* fertilizing ability.

## Materials and Methods

### Reagents

Unless otherwise stated, all reagents used in this study were of analytical grade and acquired from Sigma (Merck, Darmstadt, Germany). Fluorochromes were purchased from Thermo Fisher Scientific (Waltham, MA, USA).

### Animals and Samples

Semen samples were acquired from a local artificial insemination (AI) center (Grup Gepork S.L., Masies de Roda, Spain), which follows the ISO certification (ISO-9001:2008). The AI center performed all the procedures that involved animals in accordance with the EU Directive 2010/63/EU for animal experiments; the Animal Welfare Law issued by the Regional Government of Catalonia, Spain; and the current regulation on Health and Biosafety issued by the Department of Agriculture, Livestock, Food and Fisheries, Regional Government of Catalonia, Spain. Ejaculates from healthy and sexually mature Pietrain boars (1-3 years old) were collected using the hand-gloved method. Samples were immediately diluted to a final concentration of 33×10^6^ sperm/mL using a commercial extender (Vitasem LD, Magapor S.L., Zaragoza, Spain) and stored at 17°C until use.

For epididymal sperm samples, four healthy boars were slaughtered in a commercial slaughterhouse for genetic replacement reasons. Once slaughtered, the epididymis was collected and transported in insulated container at 5°C to our laboratory. Epididymal sperm were then flushed by placing a needle in the ductus deferens and retrogradely infusing air. The luminal fluid was collected at a section between corpus-cauda limit. The fluid from the two epididymes of each boar was pooled and was microscopically evaluated to confirm that more than 75% of sperm were viable (SYBR-14/PI staining).

No animal was manipulated by the authors, as ejaculated semen samples were acquired from a local farm (AI-center) and the abattoir donated the epididymis of boars that were sacrificed for culling reasons. No permission from an Ethics Committee was, therefore, required.

### Experimental Design

First, the presence of AKR1B1 was assessed in epidydimal and ejaculated sperm with the objective to elucidate whether this protein is acquired during ejaculation from SP. To this end, the epididymis (n=4) was flushed and the epididymal fluid was centrifuged twice (3,000×g and room temperature for 5 min) to harvest epididymal sperm. The resulting pellet was lysed to determine the levels of AKR1B1 in epididymal sperm with the Western Blot assay. On the other hand, ejaculated semen samples (n=4) were centrifuged twice (3,000 ×g and room temperature for 5 min), and the sperm pellet was lysed to determine AKR1B1 levels in ejaculated sperm also through Western Blotting.

Second, the relationship between sperm AKR1B1 levels and several sperm quality and functionality parameters were investigated. For these experiments, commercial semen samples (n=15) were split into three aliquots. The first aliquot was used to assess initial sperm quality and functionality parameters immediately after semen samples arrived at the laboratory (0 h). The second aliquot was used to evaluate sperm quality and functionality parameters after liquid storage at 17°C for 72 h. Finally, the third aliquot was centrifuged twice (3,000 ×g and room temperature for 5 min) to obtain the pellet, which was stored at -80°C until the relative content of AKR1B1 in sperm was determined.

Third, the relationship between sperm AKR1B1 and fertilizing ability was evaluated through *in vitro* fertilization using the semen samples from 24 boars (n = 24).

### Western Blot

The immunoblotting assay was used to determine the presence of AKR1B1 in ejaculated and epididymal sperm and to quantify the relative AKR1B1 content in the different sperm samples. In all cases, proteins were extracted from samples using xTractor lysis buffer (xTractor^®^ Buffer; Takara Bio, Mountain View, CA, USA), supplemented with 1% protease inhibitor, 0.1 M phenylmethylsulfonyl fluoride and 700 mM orthovanadate. Samples were incubated for 30 min on ice, with vortexing every 5 min, and then sonicated three times with five pulses. Once sonicated, they were centrifuged at 12,000 ×g and 4°C for 20 min. Supernatants were collected in siliconized Eppendorf tubes and stored at -80°C until protein quantification. Protein quantification was carried out in triplicate using a detergent compatible (DC) method (Bio-Rad; Hercules, CA, United States) and an Epoch Microplate Spectrophotometer (BioTek^®^; Winooski, VT, USA). All samples were adjusted to a final concentration of 2.5 µg/µL of total protein with the lysis buffer.

A total of 20 µg of protein was mixed (1:1, v:v) with 4× Laemmli Reducer supplemented with 5% (v:v) β-mercaptoethanol (Bio-Rad) and subsequently heated at 95°C for 7 min. A final volume of 16 µL was loaded onto 8-16% gradient gels (Mini-Protean^®^, TGX Stain-Free™ Precast Gels, Bio-Rad), and electrophoresis was conducted at 150 V for 2 h. Afterwards, proteins were transferred onto a polyvinylidene difluoride membrane (Bio-Rad) using a Trans-Blot^®^ Turbo™ system (Bio-Rad). For total protein quantification, membranes were exposed to 180 sec of UV and then read using a G:BOX Chemi XL system (SynGene; Frederick, MD, USA). Following this, membranes were blocked using blocking buffer (10 mmol/L Tris, 150 mmol/L NaCl, 0.05% Tween-20 and 5% bovine serum albumin [BSA]; pH = 7.3) for 1 h under agitation. Next, membranes were incubated with an AKR1B1 primary antibody (1:1,000 diluted in blocking buffer; ref. HPA026425, Prestige Antibodies, Merck; Germany) at 4°C overnight with agitation. In order to determine the specificity of the primary antibody, one membrane was co-incubated with the AKR1B1 blocking peptide (ref. APREST77862, Prestige Antibodies, Merck) 20 times more concentrated than the antibody. On the next day, membranes were washed thrice with 1× TBS Tween 20 (10 mmol/L Tris, 150 mmol/L NaCl, and 0.05% Tween-20; pH = 7.3) before incubation with an anti-rabbit secondary antibody conjugated with HRP (1:2,000 diluted in blocking buffer; ref. P0448, Merck) for 1 h with agitation. Finally, prior to visualization of bands, blots were washed six times (5 min each) with 1× TBS Tween 20. Detection was performed after incubation of membranes with a chemiluminescent substrate (Immobilon™ Western Detection Reagents, Millipore, United States) for 5 min, and scanning with a G:BOX Chemi XL 1.4 system (Syngene, Cambridge, UK). In all blots, two specific bands (36 kDa and 80 kDa) were observed.

Image Studio Lite v5.2.5 software (LICOR, Lincoln, NE, USA) was used for image analysis of the resulting blots. For each blot, the background level was subtracted from the density of 36 kDa and ~80 kDa bands. Moreover, each band was normalized by dividing its value with background levels. Finally, the resulting band intensity was also divided with the total protein quantity of each sample. Three technical replicates per sample were evaluated.

While the molecular weight of the monomeric AKR1B1 form is 36 kDa, the identity of the ~80 kDa band was investigated through an additional immunoblotting assay. Two pools (5 ejaculates each; one ejaculate per boar) of sperm lysates were incubated (1:1, v:v) with 16 M urea at room temperature for 1 h. Next, samples were subjected to electrophoresis and Western Blot following the previously described protocol.

### Evaluation of Sperm Quality and Functionality

#### Sperm Motility

A computer-assisted sperm analysis (CASA) system was used to assess sperm motility using an Olympus BX41 microscope (Olympus; Tokyo, Japan) with a negative phase contrast field (Olympus 10 X 0.30 PLAN objective, Olympus) connected to a computer running the ISAS software (Integrates Sperm Analysis System V1.0; Proiser S.L.; Valencia, Spain). Before motility analysis, samples were incubated at 38°C for 15 min. To examine sperm motility, 3 µL of each sample was placed into a prewarmed (38°C) Leja20 counting chamber (Leja Products BV; Nieuw-Vennep, The Netherlands). Two technical replicates, with at least 500 sperm per replicate, were counted.

Eight sperm velocity parameters were recorded: straight line velocity (VSL), average path velocity (VAP), curvilinear velocity (VCL), amplitude of lateral head displacement (ALH), beat-cross frequency (BCF), percentage of linearity (LIN), percentage of straightness (STR) and motility parameter wobble (WOB). Total motility and progressive motility were also recorded. Sperm were considered motile when VAP was ≥ 10 µm/s, and progressively motile when STR was over 45%.

#### Sperm Morphology

Sperm morphology was examined in semen samples diluted (1:1, v:v) with 0.12% formaldehyde saline solution (PanReac AppliChem; Darmstadt, Germany); a phase-contrast microscope at 1,000× magnification was used (Nikon Labophot; Nikon; Tokio, Japan). A total of 200 sperm cells were counted and those without morphology aberrations were considered as normal. Moreover, secondary alterations including sperm with proximal and distal cytoplasmic droplets and sperm with folded tails were recorded (27).

#### Flow Cytometry Assessment

Sperm viability, intracellular calcium levels and acrosome membrane integrity were assessed using a Cytoflex cytometer (Beckman Coulter; Fullerton, CA, USA). Semen samples were diluted (4×10^6^ sperm/mL) in phosphate buffered saline (1× PBS) prior to staining sperm. Briefly, sperm viability was evaluated using SYBR-14 and propidium iodide (PI), where SYBR-14 stains the nuclei of all sperm and PI only stains those of sperm that have lost their plasma membrane integrity (28). Intracellular calcium levels were evaluated through Fluo3/PI staining (29). Fluo3-AM is a non-fluorescent, non-polarized membrane-permeable dye that exhibits green fluorescence when binds to calcium (30). Acrosome membrane integrity was assessed using fluorescein-conjugated peanut agglutinin (PNA), which is a lectin that binds to the inner leaflet of the outer acrosomal membrane (31). Finally, mitochondrial membrane potential was evaluated using 5,5’,6,6’-tetrachloro-1,1’,3,3’tetraethyl-benzimidazolylcarbocyanine iodide (JC-1), that aggregates in the presence of high mitochondrial membrane potential and emits orange fluorescence (32). Throughout all the experiment, flow rate, laser voltage and sperm concentration remained unchanged. Forward scatter (FSC) and side scatter detectors (SSC) were utilized to identify sperm cells from debris events. For each sample, three technical replicates containing at least 10,000 sperm were evaluated. The CytExpert software (Ver, 2.3, Beckman Coulter) was used to analyze flow cytometry data.

Sperm viability was evaluated using the LIVE/DEAD sperm viability kit (Molecular Probes, Eugene, OR, USA), following the protocol of Garner and Johnson (1995) with minor modifications (28). Briefly, sperm were stained with SYBR-14 (final concentration: 32 nM) and PI (final concentration: 7.5 µM) at 38°C in the dark for 15 min, and subsequently analyzed with a CytoFLEX cytometer (Beckman Coulter; Fullerton, CA, USA). SYBR-14 fluorescence was detected by the fluorescein isothiocyanate (FITC) channel (525/40), and that of PI through the PC5.5 channel (690/50). Both fluorochromes were excited with a 488-nm laser, and no spill compensation was applied. The percentage of viable sperm (SYBR-14^+^/PI^-^) was recorded and used for the subsequent statistical analysis.

Sperm intracellular calcium levels were evaluated following the protocol set by Harrison et al. (1993, 29). Briefly, sperm were double stained with a solution of Fluo3-AM (final concentration: 1.2 µM) and PI (final concentration: 5.6 µM) at 38°C for 10 min. Fluorescence from Fluo3 was detected through the FITC channel (525/40). Four sperm populations were identified in dot-plots: i) viable sperm with low levels of intracellular calcium (Fluo3^-^/PI^-^); ii) viable sperm with high levels of intracellular calcium (Fluo3^+^/PI^-^); iii) non-viable sperm with low levels of intracellular calcium (Fluo3^-^/PI^+^); and iv) non-viable sperm with high levels of intracellular calcium (Fluo3^+^/PI^+^). The percentage of viable sperm with high intracellular calcium (Fluo3^+^/PI^-^) and the mean of Fluo3 fluorescence intensity per sperm were recorded and used for the subsequent statistical analysis.

Acrosome membrane integrity was evaluated using PNA-FITC/PI following the protocol set by Nagy et al. (2003, 31). Briefly, sperm were double stained with PNA conjugated with FITC (final concentration: 1.2 µM) at 38°C for 5 min in the dark. Next, sperm were stained with PI (final concentration: 5.6 µM) at 38°C for 5 min in the dark. PNA-FITC was detected by the FITC channel (525/40). Four sperm populations were observed: i) viable membrane-intact sperm (PNA-FITC^-^/PI^-^); ii) non-viable sperm having a damaged plasma membrane and an outer acrosome membrane not completely intact (PNA-FITC^+^/PI^+^); iii) non-viable sperm with a damaged plasma membrane and a completely lost outer acrosome membrane (PNA-FITC^-^/PI^+^); iv) viable sperm with a damaged plasma membrane (PNA-FITC^+^/PI^-^). The percentage of viable sperm with an intact acrosome membrane (PNA-FITC^-^/PI^-^) was recorded and used for the subsequent statistical analysis.

Mitochondrial membrane potential was evaluated with JC-1 following the protocol from Ortega-Ferrusola et al. (2008, 32). In brief, samples were incubated with JC-1 (final concentration: 750 nmol/L) at 38°C for 30 min in the dark. High mitochondrial membrane potential causes JC-1 aggregation, which results in orange fluorescence emission collected through the PE channel. On the contrary, JC-1 remains as a monomer in the presence of low mitochondrial membrane potential, emitting green fluorescence that is collected through the FITC channel. Three sperm populations were, therefore, distinguished: i) sperm with low mitochondrial membrane potential (green-stained); (ii) sperm with high mitochondrial membrane potential (orange-stained); and (iii) sperm with heterogeneous mitochondria (green and orange-stained in the same cell).

### Oocyte Maturation and *In Vitro* Fertilization

Ovaries were obtained from pre-pubertal gilts slaughtered at a local abattoir (Frigorífics Costa Brava; Riudellots de la Selva, Girona) and transported to the laboratory in 0.9% NaCl supplemented with 70 µg/mL kanamycin at 38°C. Cumulus-oocyte complexes (COC) were collected from follicles and only COCs with complete and compact cumulus mass were selected and washed in Dulbecco’s PBS (Gibco, ThermoFisher) supplemented with 4 mg/mL of BSA.

The maturation medium used was TCM-199 (Gibco) supplemented with 0.57 mM cysteine, 0.1% (w:v) PVA, 10 ng/mL EGF, 75 µg/mL of penicillin-G potassium, and 50 µg/mL of streptomycin sulfate. Groups of 40-50 COCs were transferred to a four-well multi-dish (Nunc, ThermoFisher; Waltham, MS, USA) containing 500 µL of pre-equilibrated maturation media supplemented with 10 IU/mL equine chorionic gonadotropin (eCG; Folligon; Intervet International B.V.; Boxmeer, The Netherlands) and 10 IU/mL human chorionic gonadotropin (hCG; Veterin Corion; Divasa Farmavic S.A.; Gurb, Barcelona, Spain). After 20-22 h, oocytes were transferred to 500 µL of pre-equilibrated maturation media without hormones.

Before fertilization, matured oocytes were denuded in Dulbecco’s PBS (Gibco, ThermoFisher) and placed in 50-µL drops of pre-equilibrated *in vitro* fertilization medium with 1 mM of caffeine. The basic medium used for *in vitro* fertilization was a modified Tris-buffered medium (33). Semen samples were adjusted to a final concentration of 1,000 sperm per oocyte in fertilization medium.

Oocytes and sperm were co-incubated for 5 h. The presumptive zygotes were washed and transferred (40 zygotes/well) into a four-well multi-dish containing 500 μL of NCSU23 medium (34) supplemented with 0.4% BSA, 0.3 mM pyruvate and 4.5 mM lactate. After 2 days, cleaved embryos were counted to calculate the fertilization rate; embryos were changed to NCSU23 medium supplemented with 0.4% BSA and 5.5 mM glucose, and cultured for 5 days. Embryos were classified following the Balaban & Gardner criterion (35) and the percentages of morulae, early blastocysts/blastocyst, hatching/hatched blastocysts and total embryos (sum of morulae, early blastocysts/blastocyst and hatching/hatched blastocysts) were calculated at Day 6 post-fertilization.

Oocyte maturation, *in vitro* fertilization and embryo culture were carried out at 38.5°C under a humidified atmosphere of 5% CO_2_ in air.

### Statistical Analysis

Results were analyzed using a statistical package (IBM SPSS 25.0 for Windows; Armonk, NY, USA). Data were first check for normal distribution (Shapiro-Wilk test) and homogeneity of variances (Levene test).

The immunoblotting assay revealed two specific bands at ~80 kDa and 36 kDa. The ratio between 36 kDa and ~80 kDa, and the ratio between 36 kDa and the total intensity (corresponding to the sum of the two bands) were calculated to explore the relationship between the 36 kDa band and the different parameters analyzed.

The relative content of AKR1B1 in sperm was compared between ejaculated and epididymal samples through a Mann-Whitney test. In addition, ejaculates were classified into two groups based on the median of 36 kDa/~80 kDa and 36 kDa/total ratios. Sperm quality and functionality parameters, sperm resilience to preservation (resilience ratios) and *in vitro* fertilization outcomes were subsequently compared with a Mann-Whitney test. Correlations between sperm quality and functionality parameters, and 36 kDa/~80 kDa and 36 kDa/total ratios were calculated through Spearman coefficient. The level of significance was set at *P* ≤ 0.05.

## Results

### Identification and Quantification of AKR1B1 in Epididymal and Ejaculated Sperm

**Figure 1:**
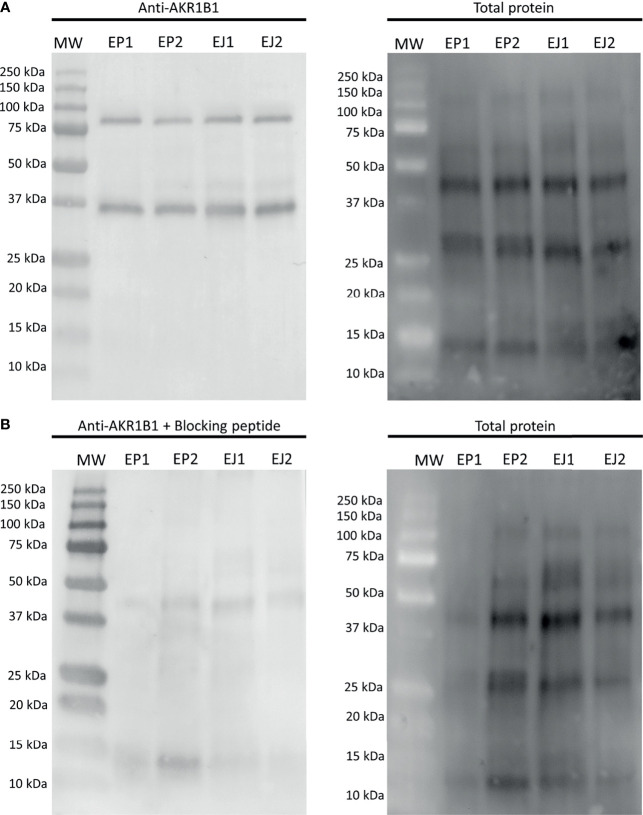
Representative Western blot analysis of **(A)** anti-AKR1B1 and **(B)** the corresponding blocking peptide in epididymal and ejaculated sperm, and their total protein controls for both membranes. MW, molecular weight; EP1 and EP2, epididymal sperm samples; EJ3 and EJ4, ejaculated sperm samples.

The first experiment aimed to evaluate whether AKR1B1 was present in epididymal and ejaculated sperm. Immunoblotting showed a double-band specific pattern for both samples at 36 kDa and ~80 kDa (**Figure 1A**). The specificity of the primary antibody was confirmed through incubating membranes with the AKR1B1 blocking peptide, as the two bands (36 kDa and ~80 kDa) disappeared (**Figure 1B**). Additionally, two pools of sperm lysates were subjected to urea denaturation to analyze whether the ~80 kDa disappeared, which would have indicated the dissociation of a potential AKR1B1 dimer. No changes, however, were observed in the band pattern between samples treated with and without urea (**Supplementary Figure 1**).

The quantification of the two bands in epididymal sperm resulted to be 0.16 ± 0.04 AU and 0.17 ± 0.05 AU for 36 kDa and ~80 kDa bands, respectively. In ejaculated sperm, the values were 0.12 ± 0.02 AU and 0.15 ± 0.04 AU for 36 kDa and ~80 kDa, respectively. No differences (*P* > 0.05) between epididymal and ejaculated sperm were found for any of the two bands (**Figure 1**).

### Relationship Between Sperm AKR1B1 Levels and Sperm Quality Parameters After 0 and 72 h of Storage at 17°C

**Figure 2:**
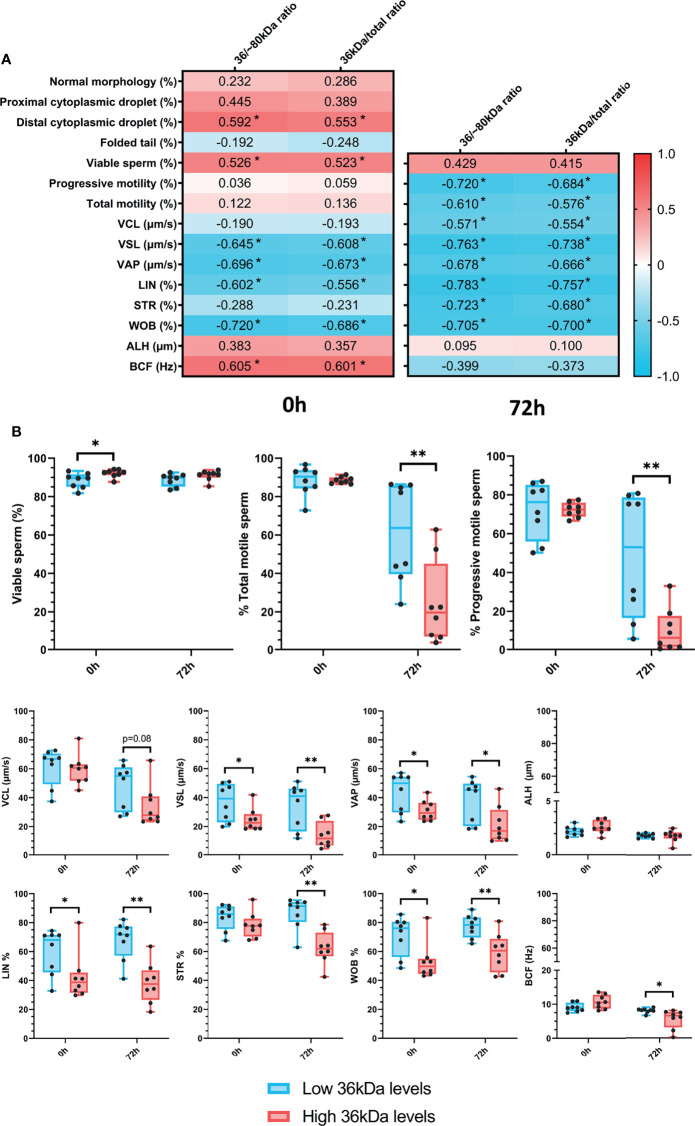
**(A)** Correlation plot of sperm quality parameters (including sperm morphology, motility and viability) and 36/~80 kDa and 36 kDa/total ratios. Semen samples of 16 AI-boars (one ejaculate per boar) were evaluated immediately after semen samples arrived at the laboratory (0 h) and after storage/preservation at 17°C for 72 h. The color saturation of red to blue represents the correlation coefficients (R) from 1 to -1, respectively. Significant correlations (*P* < 0.05) are marked with *. **(B)** Differences between groups with high and low levels of the 36 kDa band for viability and motility parameters evaluated at both 0 h and 72 h The box indicates the maximum and the minimum of each group, and the thicker line the median. Each dot represents one semen sample. Significant differences (*P* < 0.05) are marked with * and (*P* < 0.01) are marked with **.

After confirming the presence of AKR1B1 in ejaculated sperm, the potential relationship between sperm AKR1B1 levels and sperm quality parameters (in terms of sperm morphology, motility and viability) in semen samples stored for 72 h at 17°C was evaluated. To determine the relationship between the 36 kDa band and these parameters, 36 kDa/~80 kDa and 36 kDa/total ratios were calculated and used for all the subsequent analysis. Sperm quality parameters were assessed at two time-points: immediately after ejaculate collection (0 h; sperm morphology, motility and viability) and after 72 h of preservation (sperm motility and viability). Spearman correlation coefficients between sperm quality parameters, assessed at both time-points, and 36 kDa/~80 kDa and 36 kDa/total ratios of sperm AKR1B1 were calculated (**Figure 2A**).

Regarding sperm morphology, the results revealed that 36 kDa/~80 kDa and 36 kDa/total ratios were positively correlated (*P* < 0.05) with the percentage of sperm with distal cytoplasmic droplets (R = 0.592 and R = 0.553, respectively). In addition, the percentages of viable sperm were positively correlated (*P* < 0.05) with both 36 kDa/~80 kDa and 36 kDa/total ratios at 0 h (R = 0.526 and R = 0.523, respectively), but not after 72 h of liquid storage (*P* > 0.05). While no relationship between 36 kDa/~80 kDa and 36 kDa/total ratios and total and progressive sperm motility was observed at 0 h (*P* > 0.05), a negative correlation (*P* < 0.05) between these two ratios and the percentages of total (R = -0.720 and R = -0.684, respectively) and progressively motile sperm (R = -0.610 and R = -0.576, respectively) assessed after 72 h of preservation was found. In addition, 36 kDa/~80 kDa and 36 kDa/total ratios were correlated (*P* < 0.05) to kinematic parameters evaluated at both time-points. Specifically, at 0 h, 36 kDa/~80 kDa and 36 kDa/total ratios were negatively correlated (*P* < 0.05) with VSL, VAP, LIN and WOB (VSL: R = -0.645 and R = -0.608; VAP: R = -0.696 and R = -0.673; LIN: R = -0.602 and R = -0.556; WOB: R = -0.702 and R = -0.686, respectively), and positively correlated (*P* < 0.05) with BCF (R = 0.605 and R = 0.601, respectively). After 72 h of storage, all kinematic parameters, except ALH and BCF, were negatively correlated (*P* < 0.05) with 36 kDa/~80 kDa and 36 kDa/total ratios (VCL: R = -0.571 and R = -0.554; VSL: R = -0.763 and R = -0.738; VAP: R = -0.678 and R = -0.666; LIN: R = -0.783 and R = -0.757; STR: R = -0.723 and R = -0.680; WOB: R = -0.705 and R = -0.700, respectively).

Semen samples were classified into two groups according to their 36 kDa/~80 kDa and 36 kDa/total ratios of sperm AKR1B1, with high (2.3 ± 0.092 AU and 0.7 ± 0.01 AU, respectively [n = 8]) or low 36 kDa levels (1.5 ± 0.46 AU and 0.6 ± 0.08 AU, respectively [n = 8]). Then, sperm quality parameters were compared between the two groups (**Figure 2B**). In the case of sperm morphology, only the percentage of sperm with distal droplets differed between groups (*P* < 0.05), being greater in the high than in the low 36 kDa levels group (3.1 ± 1.74% *vs*. 1.2 ± 1.42%, respectively). In addition, sperm viability only differed (*P* < 0.05) at 0 h, being greater in the group with high than in that with low 36 kDa levels (92.2 ± 2.12% *vs*. 88.4 ± 3.91%, respectively). As far as sperm motility is concerned, the percentages of total and progressively motile sperm after 72 h of liquid storage were larger in the low than in the high 36 kDa levels group (total sperm motility: 61.3% ± 25.97% *vs*. 24.4% ± 21.93%; progressive sperm motility: 48.5% ± 32.49% *vs*. 10.1% ± 11.45%, respectively). Moreover, VSL, VAP, LIN and WOB were significantly higher (*P* < 0.05) in the group with low than in that with high levels of 36 kDa at both evaluation time-points (VSL: 36.9 ± 13.02% *vs*. 24.7 ± 8.02% at 0 h and 34.2 ± 15.51% *vs*. 13.9 ± 8.98% at 72 h; VAP: 43.8 ± 13.49% *vs*. 31.2 ± 7.02% at 0 h and 38.2 ± 14.88% *vs*. 21.5 ± 12.74% at 72 h; LIN: 60.0 ± 15.63% *vs*. 42.5 ± 16.22% at 0 h and 67.8 ± 13.68% *vs*. 38.2 ± 14.13% at 72 h; WOB: 70.9 ± 13.64% *vs*. 53.08 ± 13.03% at 0 h and 77.5 ± 8.02% *vs*. 59.4 ± 13.15% at 72 h, respectively). On the other hand, STR and BCF only differed (*P* < 0.05) between groups after 72 h of preservation, displaying higher values in the low than in the high 36 kDa levels group (STR: 86.7 ± 11.08% *vs*. 62.5 ± 11.36%; BCF: 8.1 ± 0.70% *vs*. 5.6 ± 2.86%, respectively).

### Relationship Between Sperm AKR1B1 Levels and Sperm Functionality Parameters After 0 h and 72 h of Preservation

**Figure 3:**
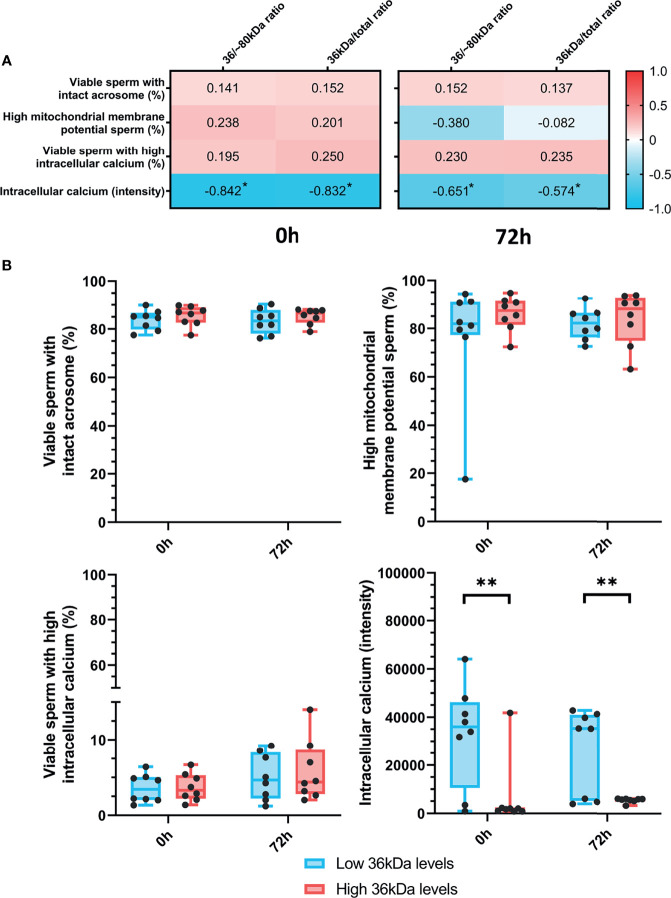
**(A)** Correlation plot of sperm functionality parameters (including acrosome integrity, mitochondrial membrane potential and intracellular calcium) and 36/~80 kDa and 36 kDa/total ratios. Semen samples of 16 AI-boars (one ejaculate per boar) were evaluated immediately after semen samples arrived at the laboratory (0 h) and after storage/preservation at 17°C for 72 h. The color saturation of red to blue represents the correlation coefficients (R) from 1 to -1, respectively. Significant correlations (*P* < 0.05) are marked with *. **(B)** Differences between groups with high and low levels of the 36 kDa band for the functionality parameters evaluated at both 0 h and 72 h. The box indicates the maximum and the minimum of each group and the thicker line the median. Each dot represents one semen sample. Significant differences (*P* < 0.01) are marked with **.

The relationship between sperm AKR1B1 (36 kDa/~80 kDa and 36 kDa/total ratios) and sperm functionality parameters (acrosome integrity, mitochondrial membrane potential and intracellular calcium levels) after 0 h and 72 h of storage at 17°C was also investigated through Spearman correlation (**Figure 3A**). A negative correlation (*P* < 0.05) between intracellular calcium levels at both evaluation time-points and the two ratios was found (0 h: R = -0.842 and R = -0.832; and 72 h: R = -0.651 and R = -0.574, respectively). In contrast, no correlation between 36 kDa/~80 kDa and 36 kDa/total ratios and the other sperm functionality parameters was observed (*P* > 0.05).

Sperm functionality parameters were also compared between the two groups (with high or low 36 kDa levels; **Figure 3B**). Whereas intracellular calcium levels were significantly (*P* < 0.05) greater in the low than in the high 36 kDa levels group after both 0 h and 72 h of preservation (0 h: 32,716.0 ± 21,328.35 AU *vs*. 6,616.0 ± 14,258.50 AU; 72 h: 26,204.9 ± 17,784.10 AU *vs*. 5,581.8 ± 94.25 AU, respectively), no significant differences in the other variables were found.

### Relationship Between Sperm AKR1B1 Levels and the Sperm Ability to Withstand Refrigeration for 72h

**Figure 4:**
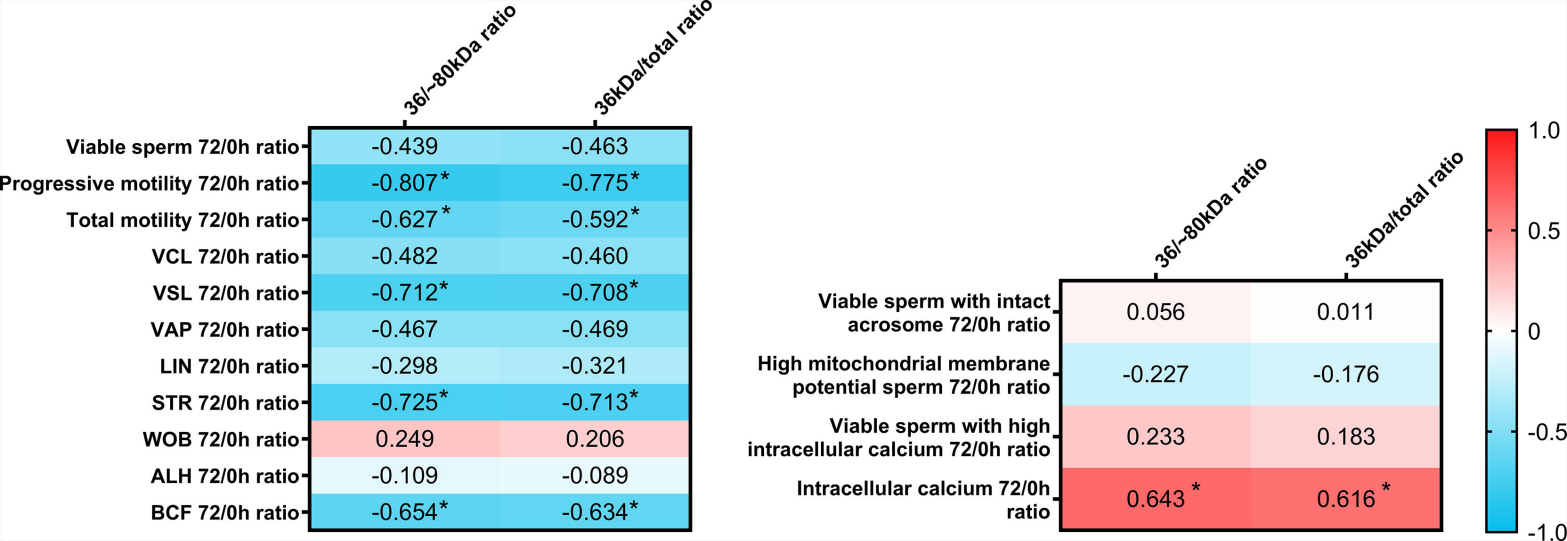
Correlation plot of sperm quality and functionality parameters (including sperm morphology, motility, viability, acrosome integrity, mitochondrial membrane potential and intracellular calcium) 72/0 h ratios and 36/~80 kDa and 36 kDa/total ratios. Semen samples of 16 AI-boars (one ejaculate per boar) were evaluated immediately after semen samples arrived at the laboratory (0 h) and after storage/preservation at 17°C for 72 h, and the ratio72/0h was calculated for each. The color saturation of red to blue represents the correlation coefficients (R) from 1 to -1, respectively. Significant correlations (*P* < 0.05) are marked with *.

The present report also aimed to evaluate whether sperm AKR1B1 could be related to the sperm resilience to preservation, as this could also be considered as an indicator of semen quality. To this end, quotients between the values of each parameter at 72 h and 0 h were calculated and defined as resilience ratios (e.g. % Progressive motility after 72 of preservation/% Progressive motility at 0 h; **Figure 4**). Regarding sperm quality parameters, Spearman correlation analysis showed that 36 kDa/~80 kDa and 36 kDa/total ratios were negatively correlated (*P* < 0.05) with resilience ratios for progressive motility (R = -0.807 and R = -0.775, respectively), total motility (R = -0.627 and R = -0.592, respectively), VSL (R = -0.712 and R = -0.708, respectively), STR (R = -0.725 and R = -0.713, respectively) and BCF (R = -0.654 and R = -0.634, respectively). Concerning sperm functionality parameters, 36 kDa/~80 kDa and 36 kDa/total ratios were positively correlated (*P* < 0.05) with resilience ratios for viable sperm with high intracellular calcium levels (R = 0.643 and R = 0.616, respectively). No other correlations were found.

In addition, resilience ratios were also compared between the two groups with high and low 36 kDa levels. Resilience ratios for progressive and total motility, VSL, STR and BCF were greater in the low than in the high 36 kDa levels group (total sperm motility: 68.6 ± 26.03% *vs*. 27.8 ± 25.12%; progressive sperm motility: 63.7 ± 35.40% *vs*. 13.7 ± 15.04%; VSL: 0.89 ± 0.185 *vs*. 0.53 ± 0.233; STR: 1.04 ± 0.082 *vs*. 0.80 ± 0.135; BCF: 0.91 ± 0.150 *vs*. 0.52 ± 0.278, respectively). Furthermore, and due to the different distribution of samples between groups, the resilience ratio for sperm viability was also greater in the low than in the high 36 kDa levels group (*P* < 0.05; 1.00 ± 0.015% *vs*. 0.99 ± 0.009%, respectively). No differences between groups were found for the other sperm quality and functionality parameters (*P* > 0.05).

### Relationship Between Sperm AKR1B1 Levels and *In Vitro* Fertilizing Ability

**Figure 5:**
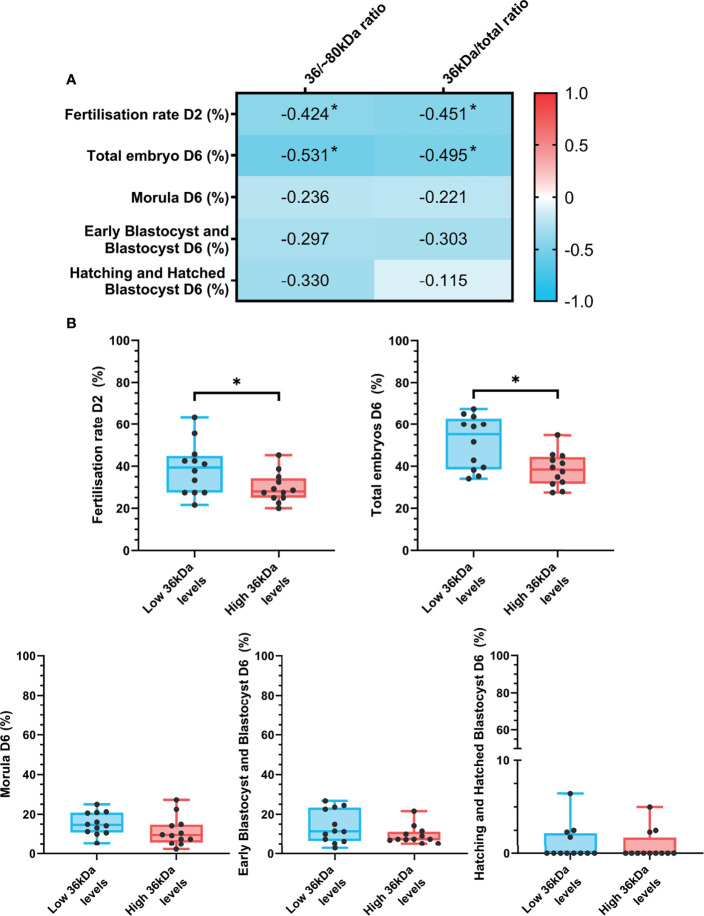
**(A)** Correlation plot of *in vitro* fertility outcomes (evaluated as fertilization rate at day 2 and percentages of total embryos, morulas, early blastocysts/blastocyst and hatching/hatched blastocysts at day 6) and 36/~80 kDa and 36 kDa/total ratios. *In vitro* fertility procedure was performed using samples from 24 AI-boars (one ejaculate per boar). The color saturation of red to blue represents the correlation coefficients (R) from 1 to -1, respectively. Significant correlations (*P* < 0.05) are marked with *. **(B)** Differences between groups with high and low levels of the 36 kDa band for *in vitro* fertility parameters. The box indicates the maximum and the minimum of each group and the thicker line the median. Each dot represents one semen sample. Significant differences (*P* < 0.05) are marked with *.

The relationship between sperm AKR1B1 levels and *in vitro* fertility outcomes was also explored in the present work. Spearman correlation coefficients between 36 kDa/~80 kDa and 36 kDa/total ratios and *in vitro* fertility parameters (fertilization rate at day 2; percentages of total embryos, morulae, early blastocysts/blastocysts and hatching/hatched blastocysts at day 6) are depicted in **Figure 5A**. Fertilization rate at day 2 negatively correlated (*P* < 0.05) with both 36 kDa/~80 kDa and 36 kDa/total ratios (R = -0.424 and R = -0.451, respectively). Similarly, a negative correlation (*P* < 0.05) between the percentage of total embryos at day 6 and the two ratios was found (R = -0.531 and R = -0.495, respectively).

*In vitro* fertility parameters were also compared between the two groups (high or low 36 kDa band levels; **Figure 5B**). Greater fertilization rate at day 2 (*P* < 0.05) was found in the group with low than in that with high 36 kDa levels (38.8 ± 12.34% *vs*. 29.7 ± 7.12%, respectively). Similarly, the percentage of total embryos at day 6 was significantly greater (*P* < 0.05) in the low than in the high 36 kDa levels group (51.4 ± 12.63% *vs*. 38.4 ± 8.09%, respectively). No differences between groups in any of the other IVF outcomes were found (*P* > 0.05).

## Discussion

Aldose Reductase B1 in SP has been proposed as a potential *in vivo* fertility marker (8), but not as a sperm quality and functionality predictor (21). In spite of this, little information regarding the role of this protein when present in sperm exists in the literature. For this reason, the present work aimed to investigate the relationship of sperm AKR1B1 with sperm quality, function and fertilizing ability using the pig as an animal model. The results of the present work showed, for the first time in mammalian species, that: i) the relative content of AKR1B1 does not differ between ejaculated and epididymal sperm; ii) the levels of the 36 kDa band detected after AKR1B1 immunoblotting are related to sperm motility and kinematic parameters; iii) sperm having greater content of the 36 kDa band show lower intracellular calcium levels; iv) the levels of the 36 kDa band are related to the sperm resilience to liquid preservation; and v) sperm with smaller content in the 36 kDa band lead to higher fertilization rate at day 2 and percentage of total embryos at day 6 post-fertilization.

The presence or activity of AKR1B1 in ejaculated sperm has been demonstrated in equine (26), bovine (17, 25) and porcine species (24). Similarly, the results of the peptide competition assay of the Western Blot showed a specific double-band pattern at 36 kDa and ~80 kDa in all ejaculated and epididymal sperm samples, thus suggesting that different forms of this protein could be physiologically present in pig sperm. In effect, this same pattern has been recently observed in the boar reproductive tract, specifically in the testis, epididymis, prostate and seminal vesicles (21), in the ovine thymus and spleen (19, 36) and in bovine peripheral blood mononuclear cells (37). After confirming that the epitope detected by the antibody was specific for AKR1B1 and not for other similar proteins, we suspected that the ~80 kDa band could correspond to a dimeric form of AKR1B1. The dimerization of both xylose reductase and AKR7, also members of the AKR superfamily, have been associated to the active form of this protein (38–41). An AKR1B1 dimer (hypothetically, the ~80 kDa band), therefore, could reasonably be assumed to be the active form of this protein, as already proposed by other authors (37). Yet, our additional experiment assessing the denaturation of the putative dimeric protein extract through urea did not confirm this hypothesis. Thus, it cannot be discarded that the ~80 kDa band corresponds to the covalent union of AKR1B1 with other proteins. In spite of this, considering that both bands are specific, our analysis of the relationship between this protein and fertility outcomes envisaged 36/~80 kDa and 36 kDa/total ratios as a measurement of the possible activation state of AKR1B1.

Aldose reductase has been reported to be transferred to sperm during epididymal maturation, as its content increases along the epididymal transit (18, 24). Yet, to the best of our knowledge, no study has addressed whether AKR1B1 levels in sperm are increased upon ejaculation due to the acquisition of this protein from the extracellular vesicles present in SP (e.g. prostasomes). The results of the immunoblotting analysis of the present work did not show differences in the relative AKR1B1 content between ejaculated and epididymal sperm, thus suggesting that the AKR1B1 contained in ejaculated sperm is mainly acquired during sperm maturation rather than at ejaculation. Remarkably, aldose reductase activity has been widely associated to epididymal maturation in bovine, murine and human species (17, 22–24). Specifically, AKR1B1 has been suggested to modulate: i) bovine and murine sperm motility through the polyol pathway (23, 25) and, ii) bovine sperm survival (25) during epididymal maturation. Considering the previous results and the existing literature, the present work evaluated if AKR1B1 levels are related to different secondary morphological abnormalities arising from an epididymal origin (27). In this regard, sperm AKR1B1 was found to be related to the percentages of sperm with distal cytoplasmic droplets, which were found to be greater when the levels of the 36 kDa band (putative inactive form of AKR1B1) were higher. It is worth mentioning that the strong genetic selection of AI-boars during the last decades has left only highly fertile individuals and, for this reason, morphological abnormalities related to inefficient epididymal maturation may not be noticeable in studies conducted in this species. The relationship between sperm AKR1B1 and the presence of distal cytoplasmic droplets, nevertheless, is in agreement with the literature (27), as higher levels of the inactive AKR1B1 form would contribute to increase the presence of morphological abnormalities originated during sperm maturation.

Besides its liaison with epididymal maturation, AKR1B1 has been found to influence ejaculated sperm physiology (24). For this reason, the present work also aimed to explore the potential relationship between the AKR1B1 and sperm functionality and *in vitro* fertilization outcomes. One of the main results of this work was that higher levels of the 36 kDa band were strongly related to lower intracellular calcium levels. Intracellular calcium is known to modulate multiple signaling pathways, the one regulating sperm motility being very relevant. In effect, increases in intracellular calcium levels are required for mammalian sperm to switch to hyperactive movement (42). Interestingly, the present work also observed a clear influence of AKR1B1 levels on several kinematic parameters. For this reason, it could be hypothesized that the participation of AKR1B1 in the regulation of intracellular calcium levels could ultimately affect sperm motility. This would be in agreement with previous studies in which AKR1B1 was found to modulate sperm motility during epididymal maturation in cattle (25) and mice (23), and during sperm capacitation in pigs (24). Remarkably, in this last study carried out in pigs, the authors found that aldose reductase was able to regulate the change from progressive to hyperactivated movement during capacitation (24). While the present work did not investigate the involvement of AKR1B1 in sperm capacitation, no relationship between acrosome integrity and AKR1B1 levels was found. For this reason, and due to the strong relationship found with intracellular calcium, which may have implications in sperm capacitation, further research is needed to clarify the precise implication of AKR1B1 in this process.

Liquid storage is widely used to preserve mammalian sperm up to 3-5 days (43). Yet, during this process there is a gradual decline of sperm quality and functionality (44, 45) and, for this reason, sperm resilience to preservation can be considered as a good semen quality and functionality indicator. Recently, our research group evaluated the potential relationship between AKR1B1 levels in pig SP and sperm quality and functionality parameters assessed after 72 h of liquid stored at 17°C, showing that the levels of this protein were not related to these parameters (21). On the contrary, the present study found that 36/~80 kDa and 36 kDa/total ratios in pig sperm are related to the sperm ability to withstand liquid storage for 72 h. Briefly, the results showed that higher levels of the potentially inactive AKR1B1 form negatively influenced the preservation of sperm motility (in terms of progressive and total motility and several kinematic parameters). These results suggest that sperm AKR1B1 might be involved in the resilience to cellular stress, evaluated here as sperm liquid storage. Although, to the best of our knowledge, no study has addressed the potential role of AKR1B1 in coping sperm stress, aldose reductases present in cattle embryos have been found to be upregulated against heat stress (46, 47). The positive effect in the resilience to this stress could be driven by the antioxidant activity of AKR1B1 (12), because this protein has already been reported to modulate ROS production at least during pig sperm capacitation (24). This hypothesis, nonetheless, should be addressed in future studies.

Considering that AKR1B1 from SP has been found to affect *in vivo* fertility outcomes (8), the present work also aimed to determine whether sperm AKR1B1 is related to *in vitro* fertilizing ability. The results showed that increased levels of the 36 kDa band (putative inactive form of AKR1B1) were negatively related to fertilization rate at day 2 and the percentage of total embryos at day 6 post-fertilization. As mentioned before, aldose reductase has already been proposed to be an essential factor for sperm function, because it modulates sperm capacitation and this could affect their fertilizing competence (24). This is supported by the findings of this work, as sperm AKR1B1 has been found to play an active role in the regulation of sperm motility. As mechanical penetration of zona pellucida is facilitated through sperm motility hyperactivation (48, 49), lower levels of the active protein could hinder oocyte penetration and, thus, fertilization. Indeed, as confirmed in the present study by day 6 observations, this lower fertilization rate is likely to result in a lower percentage of embryos. For all these reasons, it is reasonable to assume that high levels of the sperm active AKR1B1 form underlie an increased sperm fertilizing potential and, consequently, there is a positive relationship with fertility outcomes.

To conclude, aldose reductase has been widely reported to be essential for both female and male reproductive physiology. Focusing on the male, the results presented in this work showed that sperm AKR1B1 is related to epididymal maturation and modulation of sperm motility, probably through signaling pathways involving calcium homeostasis. Moreover, sperm AKR1B1 seems to have an effect on the sperm ability to withstand stress, measured in the present work as resilience to preservation. Finally, sperm AKR1B1 has also been reported to affect *in vitro* fertility outcomes, possibly through the modulation of sperm fertilizing potential. Further studies, nevertheless, are required to elucidate how AKR1B1 influences cellular stress, sperm capacitation and fertilization. Bearing in mind the current knowledge on aldose reductases, several hypotheses could be raised. First, considering that AKR1B1 can act as a detoxifying enzyme (14, 23, 39), it could exert its effect on sperm physiology through the regulation of intracellular ROS levels. Alternatively, because aldose reductase is postulated as a crucial enzyme in the polyol pathway (17, 22, 23), understanding the function of AKR1B1 in pig sperm metabolism could also help understand the relevance of that pathway in ejaculated sperm physiology and fertilizing capacity. Finally, it is likely that a balanced combination of both mechanisms promotes an optimal sperm function, thus positively influencing sperm fertilizing ability.

## Data Availability Statement

The raw data supporting the conclusions of this article will be made available by the authors, without undue reservation.

## Ethics Statement

No animal was manipulated by the authors, as ejaculated semen samples were acquired from a local farm (AI-center) and the abattoir donated the epididymis of boars that were sacrificed for genetic replacement reasons. Therefore, no permission from an Ethics Committee was required.

## Author Contributions

Conceptualization: YM-O, JR-M, and MY. Methodology: YM-O, ML, AD-B, SR, and JR-M. Formal analysis and investigation: YM-O and MY. Writing - original draft preparation: YM-O. Writing - review and editing: IB, MY, and JR-M. Funding acquisition: MY. Supervision: JR-M, IB, and MY. All authors have read and agreed to the published version of the manuscript.

## Funding

The present study was funded by the Ministry of Science and Innovation, Spain (AGL2017-88329-R and FJCI-2017-31689), the Regional Government of Catalonia, Spain (2017-SGR-1229 and 2020-FI-B-00412) and the European Commission (H2020 Research and Innovation program under the Marie Skłodowska-Curie grant agreement No 801342; Tecniospring INDUSTRY, TECSPR-19-1-0003).

## Conflict of Interest

The authors declare that the research was conducted in the absence of any commercial or financial relationships that could be construed as a potential conflict of interest.

## Publisher’s Note

All claims expressed in this article are solely those of the authors and do not necessarily represent those of their affiliated organizations, or those of the publisher, the editors and the reviewers. Any product that may be evaluated in this article, or claim that may be made by its manufacturer, is not guaranteed or endorsed by the publisher.

## Supplementary Material

Supplementary Figure 1 Western blot analysis (anti-AKR1B1 antibody) following 16 M urea denaturation of sperm samples. The control consisted of sperm samples that were not incubated with 16 M urea. MW, molecular weight; S1 and S2, sperm lysate samples; S1U and S2U, sperm lysate samples incubated with 16 M urea.
