# Supplementary figures and images for: Aldose Reductase B1 in Pig Sperm Is Related to Their Function and Fertilizing Ability
Source: Front Endocrinol (Lausanne). 2022 Jan 31;13:773249. doi: 10.3389/fendo.2022.773249 (PMC8842650; doi:10.3389/fendo.2022.773249)

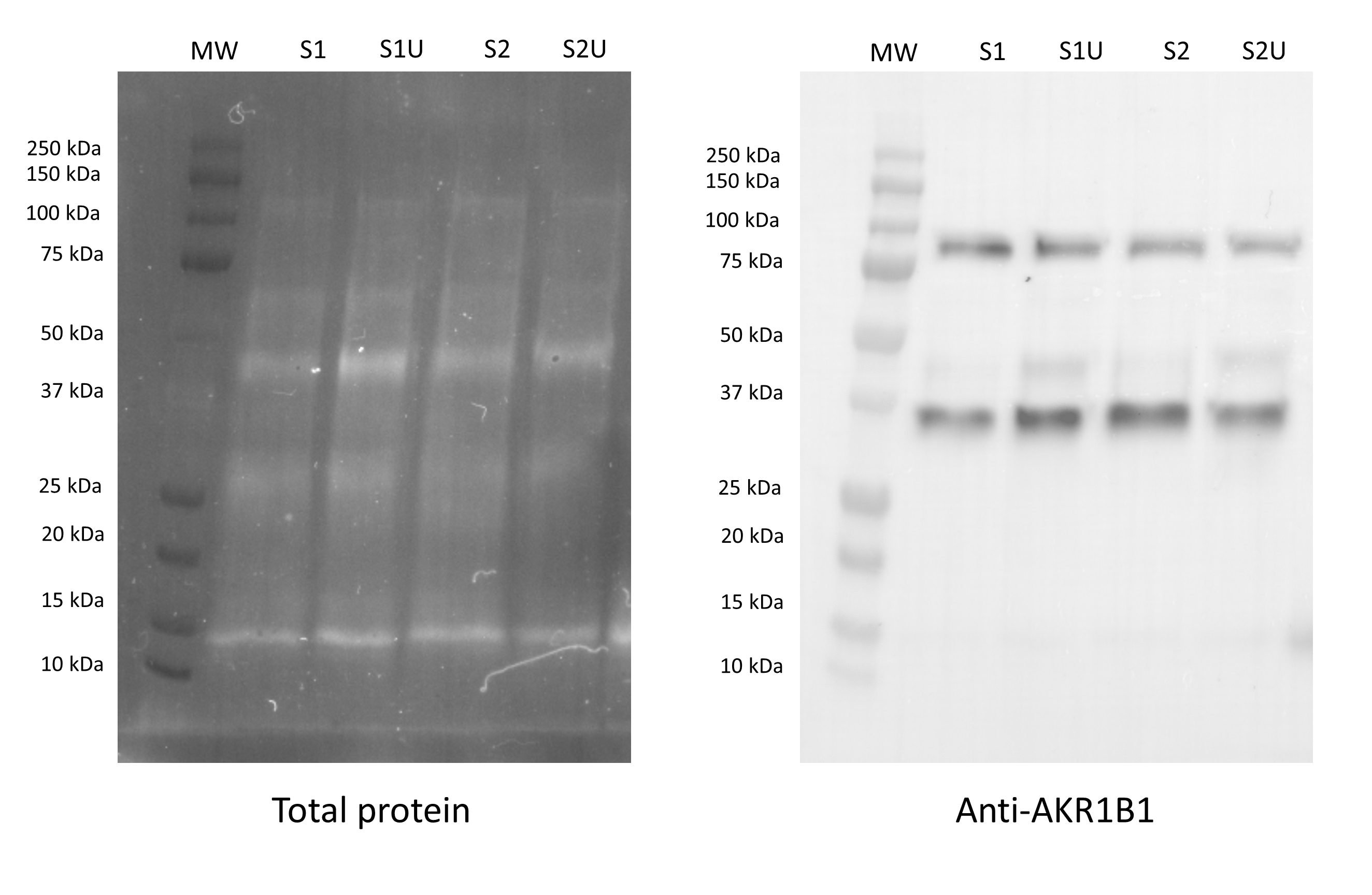

Supplement: Supplementary Figure 1 — Western blot analysis (anti-AKR1B1 antibody) following 16 M urea denaturation of sperm samples. The control consisted of sperm samples that were not incubated with 16 M urea. MW, molecular weight; S1 and S2, sperm lysate samples; S1U and S2U, sperm lysate samples incubated with 16 M urea. [file Image_1.tif]
